# Effectiveness of employer financial incentives in reducing time to report worker injury: an interrupted time series study of two Australian workers’ compensation jurisdictions

**DOI:** 10.1186/s12889-017-4998-9

**Published:** 2018-01-05

**Authors:** Tyler J. Lane, Shannon Gray, Behrooz Hassani-Mahmooei, Alex Collie

**Affiliations:** Insurance, Work and Health Group, Faculty of Medicine, Nursing and Health Sciences, Monash University, 553 St Kilda Road, Melbourne, VIC 3004 Australia

**Keywords:** Claim delays, Claim lodgement process, Workers’ compensation policy, Occupational health, Interrupted time series

## Abstract

**Background:**

Early intervention following occupational injury can improve health outcomes and reduce the duration and cost of workers’ compensation claims. Financial early reporting incentives (ERIs) for employers may shorten the time between injury and access to compensation benefits and services. We examined ERI effect on time spent in the claim lodgement process in two Australian states: South Australia (SA), which introduced them in January 2009, and Tasmania (TAS), which introduced them in July 2010.

**Methods:**

Using administrative records of 1.47 million claims lodged between July 2006 and June 2012, we conducted an interrupted time series study of ERI impact on monthly median days in the claim lodgement process. Time periods included claim reporting, insurer decision, and total time. The 18-month gap in implementation between the states allowed for a multiple baseline design. In SA, we analysed periods within claim reporting: worker and employer reporting times (similar data were not available in TAS). To account for external threats to validity, we examined impact in reference to a comparator of other Australian workers’ compensation jurisdictions.

**Results:**

Total time in the process did not immediately change, though trend significantly decreased in both jurisdictions (SA: −0.36 days per month, 95% CI −0.63 to −0.09; TAS: 0.35, −0.50 to −0.20). Claim reporting time also decreased in both (SA: −1.6 days, −2.4 to −0.8; TAS: -5.4, −7.4 to −3.3). In TAS, there was a significant increase in insurer decision time (4.6, 3.9 to 5.4) and a similar but non-significant pattern in SA. In SA, worker reporting time significantly decreased (−4.7, −5.8 to −3.5), but employer reporting time did not (−0.3, −0.8 to 0.2).

**Conclusions:**

The results suggest that ERIs reduced claim lodgement time and, in the long-term, reduced total time in the claim lodgement process. However, only worker reporting time significantly decreased in SA, indicating that ERIs may not have shortened the process through the intended target of employer reporting time. Lack of similar data in Tasmania limited our ability to determine whether this was a result of ERIs or another component of the legislative changes. Further, increases in insurer decision time highlight possible unintended negative effects.

**Electronic supplementary material:**

The online version of this article (10.1186/s12889-017-4998-9) contains supplementary material, which is available to authorized users.

## Background

Most industrialised nations have workers’ compensation (WC) or other social insurance systems to provide wage replacement, medical, and rehabilitation services in the event of occupational injury or disease. Cause-based WC systems such as those in Australia, New Zealand, Canada, and the United States provide these services after a process to determine whether the injury is compensable [1]. This can delay the claim lodgement process and access to services, which can in turn lead to more time off work [2–5], higher claim costs [4, 6, 7], and poorer long-term anxiety, depression, disability, and quality of life [8]. Further, delays at different stages in the claim lodgement process – such as initial reporting of the injury, claim lodgement, insurer liability decision, and receipt of treatment – have each been linked to more time off work [9, 10].

Providing financial incentives for employers to report worker injuries more quickly has been proposed as a way to shorten the claim lodgement process [11, 12]. With this goal in mind, two Australian WC jurisdictions, South Australia (SA) and Tasmania (TAS), introduced early reporting incentives (ERIs) in January 2009 and July 2010 respectively [13, 14]. Policies such as ERIs can have major impacts on WC claims [4, 15–17] though there has been limited research into their effect. In SA, ERIs were previously evaluated as part of a broader review of the WC legislation that introduced them, finding that they were followed by reductions in claim reporting and insurer decision times [18]. However, the analyses were largely descriptive, did not account for national trends that may confound the association, and had a limited amount of lead-in time to account for secular, or pre-existing, trends.

In this study, we addressed the following questions: 1) Were ERIs successful in reducing the duration of the claim lodgement process? And, 2) how did ERIs affect the different time periods within the claim lodgement process? We analysed administrative records of WC claims using an interrupted time series (ITS), a powerful, quasi-experimental study design of outcomes before and after an event while accounting for secular trends [19–22]. Aside from the legislative review described above, this is to our knowledge the first study to evaluate ERI impact on the claim lodgement process, account for secular and national trends, and to do so across multiple populations.

## Methods

### Setting and policy change: Early reporting incentives

In Australia, WC insurance is regulated by state, territory, and Commonwealth government agencies, each with its own policy settings and mixtures of public and private claims management systems [23]. There are nine main schemes: one for each of the six states and two territories, and one for workers in the Commonwealth government and interstate employers. In 2014, these schemes covered 10.8 million, or 94%, of Australia’s 11.5 million workers [24].

In 2007, scheme reviews for both SA [11] and TAS [12] recommended ERIs as a means of improving injured worker outcomes by reducing the time between injury and WC services. In SA, ERIs came into effect on 1 January 2009 as part of the *Workers Rehabilitation and Compensation (Scheme Review) Amendment Act 2008* [13]. From this date, employers who lodged a WC claim within two working days of becoming aware of an injury were given a rebate on their insurance excess [13], which could amount to the first 14 calendar days of wage replacement payments, or up to $2335.60 per week for two weeks [25]. As implemented in SA, ERIs were intended as a bonus to employers who performed better than the minimum required. The statutory five-day period to lodge a WC claim remained in place, enforced with a fine of up to AUD $1000 for late lodgement [26]. Eighteen months later, TAS implemented a similar set of ERIs as part of the *Workers Rehabilitation and Compensation Amendment Act 2009* [14]. From 1 July 2010, employers were required to report worker injuries to their insurer within three working days of becoming aware of them. Employers who exceeded this time became responsible for wage replacement payments for each day they were late. In TAS, ERIs were recommended partly in response to increases in the proportion of claims that were reported outside the existing statutory five-day period, reaching one-quarter by 2005/06 [12]. TAS retained a statutory five-day lodgement period with ERIs, though it was not enforced by fine [14, 27].

In both jurisdictions, ERIs were part of a larger package of WC legislative amendments. SA implemented these amendments in five waves over the course of two years beginning 1 July 2008, six months prior to ERIs coming into effect. TAS implemented all legislative amendments at one time. The features of ERIs in each jurisdiction are summarised in Table 1.

**Table 1:** Description of early reporting incentives in South Australia [51] and Tasmania [25]

	South Australia	Tasmania
Date passed parliament	19 June 2008	17 December 2009
Date effective	1 January 2009	1 July 2010
Incentive format	Rebate on employer insurance excess (first 14 calendar days of wage replacement) if employer lodges claim within two working days of becoming aware of a worker injury.	Penalty payment for wage replacement to the worker. If the employer does not report a worker injury to their insurer within three working days, they become responsible for wage replacement payments for each day they are late.
Coinciding changes in legislation (not exhaustive)	• Provisional liability granting injured workers up to 13 weeks of compensation and medical costs if a liability decision is not made within 7 days of worker report. • Changes to dispute resolution system. • Cap on what lawyers can charge in disputes and change of pay structure to remove financial incentives for lawyers to perpetuate claims. • Requirements for employers to have rehabilitation and return to work coordinators.	• Payments for counselling for families of deceased workers. • Payments for medical and other expenses for up to 12 months after the cessation of income replacement. • Increase in maximum lump sum for permanent impairment. • Extension of weekly payments for workers based on whole-person impairment. • Increases in income replacement rates and delays in step-downs. • Claimants exempted from step-downs provided they have returned to work for at least 50% of pre-injury hours or duties, or where employer refused or is unable to provide alternative duties. • Reduce whole-person impairment thresholds for access to common law damages. • Requirements for return to work and injury management plans.
Non-coinciding changes in legislation (not exhaustive)	• 1 July 2008: Reductions to income-replacement rates. Notice periods before benefit reduction or cessation. Code of workers’ rights. Establishment of WorkCover ombudsman and Return to Work Inspectorate. Permitting weekly payments in case of disputes. • 1 April 2009: Changes to work capacity reviews for claimants seeking income replacement beyond 130 weeks (2.5 years). Introduction of 5% impairment threshold for permanent injury payments in physical injury cases. Increase of pain and suffering payments to $400,000. Establishment of independent medical panels for decisions on medical questions. • 1 July 2009: Restrictions on use of redemptions (one-off payments) to finalise claims with injury date on or after 1 July 2006. • 1 July 2010: Restrictions on use of redemptions (one-off payments) to finalise claims for all claims.	• None

### Data source

The data were derived from the *National Dataset for Compensation-based Statistics*, a collection of de-identified WC claim records from each Australian WC jurisdiction, compiled by Safe Work Australia for inter-jurisdictional comparisons and national data analysis [23]. To minimise bias from co-occurring events, changes in SA and TAS were evaluated in contrast to a comparator [20, 21, 28] composed of other Australian WC jurisdictions that had not implemented ERIs and adhered to the most up-to-date coding standards for the duration of the study. These included New South Wales, Victoria, Western Australia, and the Northern Territory. Claims were eligible for inclusion if they had been lodged with an insurer between July 2006 and June 2012 and subsequently accepted, resulting in *N* = 1,470,303 records.

### Study design

We evaluated ERI impact with a multiple baseline ITS study design. ITS is considered one of the most powerful quasi-experimental designs for evaluating natural experiments like policy change or community interventions where data have been collected at regular intervals before and after a time-bounded event [19–21, 28, 29]. ITS can be applied where randomised controlled trials are cost-prohibitive, impractical, or unethical, and can evaluate population-level impacts in real-world settings [21, 22, 28–31]. Unlike other before-and-after analytical techniques such as difference-in-differences, ITS accounts for secular trends, minimising the likelihood that observed differences due to pre-existing trends are misattributed to the event [28, 31–34]. ITS also detects trend changes, such as the progressive reduction of a surgical procedure following publication of evidence that it was ineffective [35]. Often used to evaluate count or rate changes, such as the incidence of acute coronary events [19] and gun homicides [36], ITS has been applied to analysis of changes in central tendency, such as the impact of publishing guidelines on median reporting quality in peer review journal articles [37] and reimbursement caps on the mean number of prescriptions dispensed to public assistance recipients [28]. In WC settings, ITS has been used to evaluate the impact of experience-rated premium discount programmes on claim rates [38]. The 18-month gap between SA and TAS’s ERI implementation allowed for the addition of a multiple baseline design, which minimises the biasing potential of co-occurring events by analysing the same yet staggered events in different populations [22, 29, 30].

### Outcomes

**Fig. 1:**
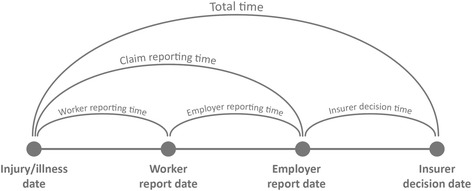
Key events and time periods in the claim lodgement process

Outcomes were monthly median days in three main time periods comprising the claim lodgement process, which were total time (injury/illness date to insurer decision date), claim reporting time (injury/illness date to employer report date), and insurer decision time (employer report date to insurer decision date). In SA, it was possible to evaluate two time periods that together comprised claim reporting time: worker reporting time (injury/illness date to worker report date) and employer reporting time (worker report date to employer report date). The time periods are illustrated in Fig. 1. We used the median for stability and to evaluate ERI impact on the “average” claim. After identifying some substantial changes to the upper range of the interquartile range (IQR) following ERI implementation, and in light of nearly 25% of claims in TAS exceeding the statutory five-day reporting period [12], we conducted sensitivity analyses with the 75th percentile as the outcome. Worker report dates were missing in 75.6% of TAS cases (41,388 of 54,765 cases), far below the recommended threshold of 80% complete data [31]. This date was also entirely missing in New South Wales, which we excluded from the comparator for these analyses. All other jurisdictions had at least 80% complete data for each outcome (see Additional file 1: Table S1 for a summary of data completeness).

### Analysis

Crude analyses describe the duration of the claim lodgement process before and after ERIs using median number of days with IQR.

For ITS analyses, we created an aggregate dataset of monthly median durations for SA, TAS, and the comparator, along with the 75th percentile for sensitivity analyses. SA and TAS were analysed in separate models, both in reference to the comparator. Changes were evaluated with a generalised least-squares regression [39]. Harmonic terms were added to the models to account for seasonality, if statistically significant [40, 41]. To account for additional autocorrelation, we fit the data to autoregressive-moving average (ARMA) models based on correlated residuals observed in Autocorrelation Function (ACF) and Partial Autocorrelation Function (PACF) plots [28, 39, 42]. For each analysis, all possible models were compared on Akaike Information Criterion (AIC) and Bayesian Information Criterion (BIC) with an analysis of variance (ANOVA). The final models, with harmonic and ARMA terms, are listed in R code in the additional files. To check for history threats to internal validity such as national-level events, we conducted an ITS analysis of monthly claim volumes. Volumes were indexed to the first point in the time series to account for large volume differences between the groups. Methods are reported against the *Quality Criteria for ITS Designs* [31] in Additional file 1: Table S1. Seasonally-adjusted ITS trend lines were plotted over monthly data points for each jurisdiction using code adapted from the ITS tutorial in Lopez Bernal et al. [19]. For visual clarity, trend lines for SA and TAS reflect regression models without reference to the comparator, and the comparator trend line includes both ERI event interruptions.

Analyses were conducted in R (v3.3.2) [43] using RStudio (v1.0.44) [44] and the nlme package for generalised least squares regression analyses [45]. R Markdown files containing code for analyses and plotting can be found in the Additional files 2, 3, 4, 5 and 6.

## Results

In crude before-and-after analyses, median durations for most time periods in the claim lodgement process were shorter after ERI implementation. The exception was insurer decision time, which increased from 7 to 8 days in SA, and from 2 to 6 days in TAS. In the comparator, insurer decision time did not change at either ERI implementation, and worker reporting time increased from 10 to 11 days at SA implementation. Further, the 75th percentile of insurer decision times increased in SA (doubling from 22 to 45 days) and TAS (tripling from 4 to 12 days), while decreasing in the comparator. The increase was reflected in total time, where the 75th percentile increased from 65 to 79 days in SA and 35 to 38 days in TAS. These results are summarised in Table 2.

**Table 2:** The impact of South Australia and Tasmania’s early reporting incentives on number of days in the claims process, in reference to a comparator consisting of other Australian workers’ compensation jurisdictions

	Median number of days (IQR)				ITS analyses			
	Pre-ERI		Post-ERI		Median level change, days (95% CI)		Median trend change, days per month (95% CI)	
Total time								
South Australia	25	(14 to 65)	23	(10 to 79)	−0.7	(−4.7 to 3.3)	−0.36**	(−0.63 to −0.09)
Comparator	25	(11 to 70)	20	(8 to 57)	0.3	(−2.5 to 3.2)	0.11	(−0.08 to 0.31)
Tasmania	19	(12 to 35)	17	(9 to 38)	−0.6	(−2.9 to 1.6)	−0.35***	(−0.50 to −0.20)
Comparator	23	(10 to 68)	18	(8 to 53)	−0.8	(−2.4 to 0.8)	0.15**	(0.05 to 0.26)
Claim reporting time								
South Australia	13	(7 to 28)	7	(2 to 19)	−1.6***	(−2.4 to −0.8)	0.01	(−0.03 to 0.06)
Comparator	11	(5 το 26)	9	(3 to 24)	−1.4***	(−2.0 to −0.8)	0.02	(−0.01 to 0.05)
Tasmania	15	(9 to 29)	9	(4 to 21)	−5.4***	(−7.4 to −3.3)	−0.32***	(−0.49 to −0.14)
Comparator	10	(4 to 26)	9	(3 to 24)	−0.3	(−1.1 to 1.8)	0.14*	(0.01 to 0.27)
Insurer decision time								
South Australia	7	(3 to 22)	8	(4 to 45)	−1.2	(−2.9 to 0.5)	−0.00	(−0.10 to 0.10)
Comparator	6	(2 to 27)	6	(2 to 20)	−0.2	(−1.4 to 1.1)	0.01	(−0.07 to 0.08)
Tasmania	2	(1 to 4)	6	(2 to 12)	4.6***	(3.9 to 5.4)	−0.00	(−0.05 to 0.05)
Comparator	6	(2 to 27)	6	(2 to 15)	−0.5	(−1.1 to 0.1)	0.04*	(0.01 to 0.08)
Worker reporting time								
South Australia	7	(2 to 20)	1	(0 to 8)	−4.7***	(−5.8 to −3.5)	−0.03	(−0.10 to 0.04)
Comparator	10	(4 to 28)	11	(4 to 29)	−0.1	(−0.9 to 0.7)	−0.01	(−0.06 to 0.04)
Employer reporting time								
South Australia	2	(0 to 6)	1	(0 to 6)	−0.3	(−0.8 to 0.2)	0.05***	(0.02 to 0.07)
Comparator	6	(2 to 10)	5	(2 to 10)	−0.3	(−0.7 to 0.0)	−0.02*	(−0.04 to −0.00)

*** *p* < .001; ** *p* < .01; *p* < .05

### Interrupted time series analyses

#### Time in the claim lodgement process

Following implementation of ERIs, neither SA nor TAS exhibited a level change in total time in the claim lodgement process, though trend decreased by one-third a day per month in both (SA: -0.36, 95% CI: −0.63 to −0.09; TAS: −0.35, −0.50 to −0.20). Claim reporting times decreased in both jurisdictions (SA: −1.6 days, −2.4 to −0.8; TAS: -5.4 days, −7.4 to −3.3). Trend did not change significantly in SA, indicating the changes were sustained, though in TAS there was a decrease of 0.32 days per month (−0.49 to −0.14), indicating longer-term reductions.

**Fig. 2:**
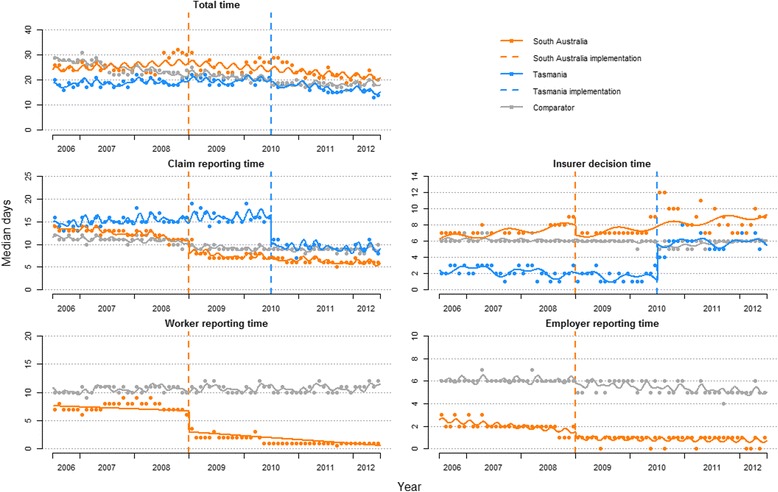
Seasonally-adjusted trends in monthly median time lags in the claim lodgement process pre- and post-early reporting incentives in South Australia and Tasmania, in reference to a comparator consisting of other Australian workers’ compensation jurisdictions, July 2006 to June 2012

In SA, analysis of the time periods within claim reporting time found worker reporting time decreased 4.7 days (−5.8 to −3.5), though there was no significant level change in employer reporting time, the ERI target (−0.3, −0.8 to 0.2). There was a marginal though significant trend increase (0.05, 0.02 to 0.07), though the plot suggests this was the secular trend levelling off after approaching zero (see Fig. 2). Median employer reporting time may not have been sensitive to ERIs, which was two calendar days pre-ERI (the threshold in SA was two working days, which would be longer than median employer reporting time on average due to weekends and holidays). Therefore, we report the 75th percentile of claims, where pre-ERI was 6 days. In this case, worker reporting time saw a significant *increase* in level (1.4, 0.7 to 2.2), and trend (0.08, 0.04 to 0.12).

In TAS, the decrease in claim reporting time was countered by a 4.6 day increase in insurer decision time (3.9 to 5.4); there was no significant post-ERI trend change, indicating the change was sustained. Though insurer decision time did not change significantly in SA, there was a substantial increase in insurer decision time six months prior to ERI implementation, coinciding with the first wave of SA’s WC amendments in July 2008. In the year prior to the first implementation wave (July 2007 to June 2008), the range of median insurer decision days was 6–7, which increased to 8–9 in the six months prior to ERI implementation (July to December 2008) (see Fig. 2). The trend was more pronounced among the 75th percentile, where the figures were 14–19 days in the year prior to the first wave and 48–63 days in the six months prior to ERI implementation (see Additional file 7: Figure S1 and Additional file 8: Table S2). Around mid-2010, there was another increase, coupled with greater variability in insurer decision time: while at 6–7 days between January 2009 and April 2010, from May 2010 onwards the range fluctuated between 7 and 12 days. These results are summarised in Table 2 and plotted in Fig. 2.

The 75th percentile of claims generally followed a similar pattern as the median. Exceptions included the differences in employer reporting time in SA, noted above. In TAS, sensitivity analyses identified two substantial differences. There was a lack of a trend change in total time among the 75th percentile (median analysis: down 0.35 days per month), and a lack of a level change in insurer decision time (median analysis: up 4.6 days). However, plotting of the trend line suggested a non-linear trend increase, which we accounted for using squared and cubed post-ERI trend terms. This model had better fit than that assuming a linear post-ERI trend (*p* < .001), and confirmed a significant trend increase in TAS. Sensitivity analysis results are summarised in Additional file 8: Table S2 and plotted in Additional file 7: Figure S1.

#### Claim volumes

Without reference to the comparator, both SA and TAS experienced significant changes to claim volumes at ERI implementation: SA was down 4.0% on the volume indexed at July 2006 (95% CI: −6.7 to −1.2%) with a significant trend increase (0.51% per month, 0.37 to 0.65%), while TAS was up 9.2% (1.6 to 16.8%) without a trend change. The comparator followed a similar pattern, with claim volumes decreasing at SA implementation (−4.8%, −7.1 to −2.4%) with a 0.21% per month trend increase (0.10 to 0.32%), while increasing at TAS implementation (8.3%, 2.9 to 13.6%). Controlling for these changes attenuated volume level changes in both SA and TAS to non-significance, though there remained a significant trend increase in SA (0.30%, 0.15 to 0.46%). These results are summarised in Additional file 9: Table S3 and plotted in Additional file 10: Figure S2.

## Discussion

Our findings suggest that ERIs achieved some success in shortening the claim lodgement process. Following implementation, median claim reporting times saw significant level reductions with either sustained or decreased trends, and median total time saw significant trend reductions, in both SA and TAS. Trend reductions in total time suggest long-term ERI effectiveness. However, sensitivity analysis of the 75th percentile found neither level nor trend reduction in total time in TAS. Part of the justification for ERIs in TAS was that nearly 25% of claims exceeded the old statutory reporting period [12]. Our findings suggest that ERIs did not improve timeliness for those it was designed to help.

There are several other issues with attributing success to the policy. The first is that in SA there was no significant decrease in the ERI target, employer reporting time. One possible explanation is that the ERI design in SA removed incentives to report an injury quickly once the two day threshold had been crossed, since employers were ineligible for the rebate after this time. It may have even reduced the sense of urgency among employers to report injuries, as demonstrated by the increase among the 75th percentile. This is in contrast to the penalty format in TAS, which accrued for each day employers were late to report an injury, building pressure once they crossed the three day threshold.

In SA, the change in claim reporting time was driven by reductions in worker reporting time. While a decrease in worker reporting time is a positive outcome, since delays in this time period are predictive of claims becoming long-duration [9, 10], the implication is that these reductions are indirectly attributable to ERIs, or to another cause entirely. One possibility is that provisional liability, which grants injured workers WC services while awaiting a decision on their claim, fostered a sense of certainty of benefits and encouraged workers to engage with the system earlier [18]. In SA, ERIs and provisional liability were part of the same amendment package and were implemented concurrently [13]. TAS also had provisional liability, though it had been introduced two decades earlier, rather than at the same time as ERIs [27]. It was unfortunate that we were unable to examine worker reporting time and employer reporting time in TAS, as the contrast between ERIs introduced in concert with provisional liability (SA) and ERIs introduced where provisional liability was established (TAS) would have been illuminating.

The second issue with attributing success to the policy is that the reductions in claim reporting time in TAS were offset by increases in insurer decision time. These may have been due to an increase in administrative burden, or the time costs for insurer and regulatory staff to learn, manage, and implement new regulations [46]. In TAS, ERIs were enforced via the transfer of wage replacement payments from insurers to employers, which may have entailed additional administrative burden for insurers on top of adapting to new policies. Interestingly, sensitivity analysis found no level change, but a significant, non-linear trend increase among the 75th percentile of claims. This suggests that whatever caused the increase in insurer decision time – whether administrative burden or some other factor – only affected longer-duration claims gradually. It is unclear why the effect would be different from the median, and merits further investigation.

Trends in SA further suggest administrative burden as the driver of increased insurer decision time. While it did not significantly change at ERI implementation, insurer decision time began to increase with the first wave of WC amendments in July 2008. The second increase in mid-2010 is likely explained by disruption due to the SA regulator’s introduction of a new IT system in late April 2010 [47]. Administrative burden may have been more of an issue in TAS, where claims were managed by smaller organisations (seven insurers for 9000 claims per annum in TAS, versus one claims agent for 27,000 claims per annum in SA [24, 25]). Insurers in TAS, which had to adopt the entire legislation at one time, likely had fewer resources to cope with the administrative burden [48]. There was also an increase in monthly claim volumes in TAS (significant when not adjusting for the comparator), which may have been more difficult for their smaller insurers to manage. However, this does not explain why TAS’s increases in median insurer decision time were sustained, nor the trend increase among the 75th percentile.

Though SA and TAS have cause-based WC systems, provisional liability made insurer decision time less of a barrier to service access. Prospective claimants in TAS were entitled to wage replacement and treatment from the moment their claim was lodged [27], while in SA they began seven days after worker report date [13]. Additionally, TAS’s amendment package introduced a requirement for employers to provide rehabilitation services as soon as they become aware of the injury [14]. With provisional liability, the decreases in claim reporting and worker reporting times following ERIs would mean accelerated access to WC services, which was the policy aim. In the absence of provisional liability, increases in insurer decision time, whether due to ERIs or the legislation they are part of, could delay WC service access. Our findings suggest that increases in insurer decision time may be due to administrative burden of large WC legislative changes. Future ERI iterations are likely to be delivered similarly, as WC changes often come in larger packages. Without a provisional liability safeguard, WC changes may delay insurer decision time and WC service access. Alternatively, provisional liability may counter the sense of “urgency” for insurers to deal with claims more quickly [18] if access to WC services is not contingent on insurer decision. However, even if service access is accelerated, increased insurer decision times could worsen outcomes for the injured worker [8].

Our findings concur with the review of SA’s WC legislation in terms of ERI impact on claim reporting time (both found a reduction), though differ on insurer decision time [18]. The review’s authors found insurer decision time decreased, which they attributed to the introduction of ERIs and provisional liability. However, this time series began in the quarter ending June 2008, which was near the first wave of SA’s legislative amendments and, according to our data, was the start of a period of increased insurer decision time. The report thus started at a higher baseline, creating the perception that later reductions were a decline rather than a regression to the mean.

### Strengths and limitations

This study’s main strengths were the use of a powerful quasi-experimental research design, the ITS, with multiple baselines, a comparator, and adjustments for seasonality and autocorrelation, using a large number of claims from a dataset created for inter-jurisdictional comparisons and national data analysis [23]. The number of observations exceeded most recommended minimums and generated greater certainty about seasonal and autocorrelation adjustments. Additionally, we evaluated several time periods within the claim lodgement process, exploring both the ERI target, claim reporting times (via employer reporting times), and areas that could have been indirectly affected (insurer decision time).

The study had several limitations. The ITS design is vulnerable to confounding from co-occurring events, which can lead to misattributions of cause [29, 32]. Further, ITS assumes linear trends among data, which is likely to be violated with longer time series [32, 33], and with the use of aggregated data, does not allow for adjustment of individual-level characteristics [28, 33]. The staggered introduction of SA’s amendments likely confounded insurer decision time, as did the introduction of a new IT system.

Changes in monthly claim volumes raise the possibility of selection bias, but also demonstrate the strength of using a comparator within an ITS study design. While claim volumes significantly changed in SA and TAS following ERI implementation, both attenuated to non-significance when adjusting for the comparator (i.e., controlling for the national-level effect). The timing and direction of volume changes suggest they were the result of the Global Financial Crisis (GFC). Though the GFC did not have as large an impact on Australia as other developed economies [49], there was nevertheless a reduction in WC claims. This may be due to worker reluctance to make a claim and risk unemployment in the face of economic uncertainty, and/or employers favouring experienced, less risky workers in hiring/firing decisions, resulting in safer worker cohorts [50]. If the change in claim volumes was due to the GFC, use of the comparator likely reduced its potential to bias outcomes.

The comparator was unable to adjust for all co-occurring events, particularly those specific to each jurisdiction. While SA and TAS introduced ERIs as part of a larger set of legislative WC amendment packages, they likely varied in terms of their impact on the claim lodgement process. For instance, the staggered introduction of WC amendments in SA likely diffused administrative burden. The introduction of provisional liability in concert with ERIs in SA could have incentivised injury reporting among workers due to greater certainty of WC services. There is also the possibility that other co-occurring events, specific to either jurisdiction, could have confounded the associations. Further, the two ERI policies had different designs. It is unclear whether the number of days prescribed (two in SA, three in TAS) or the incentive type (limited rebate in SA, accruing penalty in TAS) modified ERI effectiveness. Lastly, in several cases baseline durations were different, which likely moderated ERI impact.

## Conclusions

After implementing ERIs, claim reporting time in two Australian WC jurisdictions decreased. This suggests the policy succeeded in reducing one source of delay in the claim lodgement process. However, where it could be evaluated, we did not find a significant effect on the ERI target, employer reporting time, which raises questions about why the reductions occurred. Increases in insurer decision time highlight the possibility of negative indirect effects of ERIs, or the legislation they are part of. SA and TAS both had provisional liability safeguards that removed insurer decision time as a barrier to WC service access, though this may not be the case in other jurisdictions considering ERIs. While we evaluated the impact of ERIs on the claim lodgement process, the ultimate aim was to improve injured worker outcomes such as health, disability duration, and claim costs. We recommend these areas for future research into ERI impact.

## Additional files

Additional file 1: Table S1. Responses to the Quality Criteria for ITS Designs in Ramsay et al. 2003 [31]. Word document with table, citations, and bibliography. (DOCX 24 kb)

Additional file 2: Main analyses R code, main analyses. R Markdown file. (RMD 16 kb)

Additional file 3: Main plots R code, main plots. R Markdown file. (RMD 26 kb)

Additional file 4: Sensitivity analyses. R code, sensitivity analyses. R Markdown file. (RMD 19 kb)

Additional file 5: Sensitivity plots R code, sensitivity plots. R Markdown file. (RMD 28 kb)

Additional file 6: Claim volumes analysis. R code, claim volumes analysis and plot. R Markdown file. (RMD 15 kb)

Additional file 7: Figure S1. Seasonally-adjusted trends in monthly 75th percentile time lags in the claims process pre- and post-early reporting incentives in South Australia and Tasmania, in reference to a comparator consisting of other Australian workers’ compensation jurisdictions, July 2006 to June 2012. .png image containing plots. (PNG 20 kb)

Additional file 8: Table S2. The impact of South Australia and Tasmania’s early reporting incentives on number of days in the claims process, in reference to a comparator consisting of other Australian workers’ compensation jurisdictions (sensitivity analysis with 75th percentile). Word document with table. (DOCX 15 kb)

Additional file 9: Table S3. Monthly counts of workers’ compensation claims pre- and post-early reporting incentives in South Australia and Tasmania, in reference to a comparator consisting of other Australian workers’ compensation jurisdictions, July 2006 to June 2012. Word document with table. (DOCX 15 kb)

Additional file 10: Figure S2. Seasonally-adjusted trends in monthly claim volumes (indexed to July 2006), pre- and post-early reporting incentives in South Australia and Tasmania, in reference to a comparator consisting of other Australian workers’ compensation jurisdictions, July 2006 to June 2012. .png image containing plot. (PNG 14 kb)

## Abbreviations

ARMA: Autoregressive Moving Average
ERI: Early Reporting Incentive
GFC: Global Financial Crisis
IQR: Inter-Quartile Range
ITS: Interrupted Time Series
SA: South Australia
TAS: Tasmania
WC: Workers’ Compensation

## References

[1] Lippel K, Lötters F. Public insurance systems: A Comparison of Cause-Based and Disability-Based Income Support Systems. In: Loisel P, Anema JR, editors.. Manag. New York, NY: Springer; 2013. p. 183–202.

[2] Kucera KL, Lipscomb HJ, Silverstein B, Cameron W (2009). Predictors of delayed return to work after back injury: a case-control analysis of union carpenters in Washington state. Am J Ind Med.

[3] Lusted M (1993). Predicting return to work after rehabilitation for low back injury. Aust J Physiother Australian Physiotherapy Association.

[4] Shraim M, Cifuentes M, Willetts JL, Marucci-Wellman HR, Pransky G (2015). Length of disability and medical costs in low back pain: do state workers’ compensation policies make a difference?. J Occup Environ Med.

[5] Stover B, Wickizer TM, Zimmerman F, Fulton-Kehoe D, Franklin G (2007). Prognostic factors of long-term disability in a workers’ compensation system. J Occup Environ Med.

[6] Gallagher RM, Myers P (1996). Referral delay in back pain patients on worker’s compensation. Psychosomatics Elsevier.

[7] Pitruzzello G-R. The high cost of delays: findings on a lag-time study. Issues Rep. 2000:13–7.

[8] Grant GM, O’Donnell ML, Spittal MJ, Creamer M, Studdert DM (2014). Relationship between stressfulness of claiming for injury compensation and long-term recovery. JAMA Psychiatry.

[9] Besen E, Harrell M, Pransky G (2016). Lag times in reporting injuries, receiving medical mare, and missing work: associations with the length of work disability in occupational back injuries. J Occup Environ Med.

[10] Cocker F, Sim MR, Kelsall H, Smith PM. Injury reporting, employer lodgement and compensation payment delays and RTW outcomes in long-term injured workers. Under Rev.

[11] Clayton A, Walsh J (2007). Review of the south Australian workers’ compensation system report.

[12] Clayton A (2007). Review of the Tasmanian workers compensation system.

[13] Parliament of South Australia. Workers Rehabilitation and Compensation (Scheme Review) Amendment Act 2008. 2008.

[14] Parliament of Tasmania. Workers Rehabilitation and Compensation Amendment Act. 2009.

[15] Anema JR, Schellart AJM, Cassidy JD, Loisel P, Veerman TJ, Van Der Beek AJ (2009). Can cross country differences in return-to-work after chronic occupational back pain be explained? An exploratory analysis on disability policies in a six country cohort study. J Occup Rehabil.

[16] Cassidy JD, Carroll LJ, Côté P, Lemstra M, Berglund A, Nygren Å (2000). Effect of eliminating compensation for pain and suffering on the outcome of insurance claims for whiplash injury. N Engl J Med.

[17] Collie A, Lane TJ, Hassani-Mahmooei B, Thompson J, McLeod C (2016). Does time off work after injury vary by jurisdiction? A comparative study of eight Australian workers’ compensation systems. BMJ Open.

[18] Cossey B, Latham C (2011). Review of the impact of the workers rehabilitation and compensation (scheme review) amendment act 2008.

[19] Lopez Bernal J, Cummins S, Gasparrini A, Bernal JL, Cummins S, Gasparrini A (2017). Interrupted time series regression for the evaluation of public health interventions: a tutorial. Int J Epidemiol.

[20] Penfold RB, Zhang F (2013). Use of interrupted time series analysis in evaluating health care quality improvements. Acad Pediatr Elsevier.

[21] Shadish WR, Cook TD, Campbell DT (2002). Experimental and quasi-experimental designs for generalized causal inference.

[22] Schelvis RMC, Oude Hengel KM, Burdorf A, Blatter BM, Strijk JE, van der Beek AJ (2015). Evaluation of occupational health interventions using a randomized controlled trial: challenges and alternative research designs. Scand. J. Work Environ Health.

[23] Safe Work Australia. National Data Set for Compensation-based Statistics Third Edition. Canberra, AU; 2004.

[24] Lane TJ, Collie A, Hassani-Mahmooei B (2016). Work-related injury and illness in Australia, 2004 to 2014.

[25] Safe Work Australia. Comparison of Workers’ Compensation Arrangements in Australia and New Zealand 2010. Safe Work Aust. Canberra, AU; 2010.

[26] Parliament of South Australia. Workers Rehabilitation and Compensation Act 1986. 1986.

[27] Parliament of Tasmania. Workers Rehabilitation and Compensation Act 1988. 1988.

[28] Wagner AK, Soumerai SB, Zhang F, Ross-Degnan D (2002). Segmented regression analysis of interrupted time series studies in medication use research. J Clin Pharm Ther.

[29] Biglan A, Ary D, Wagenaar AC (2000). The value of interrupted time-series experiments for community intervention research. Prev Sci.

[30] Hawkins NG, Sanson-Fisher RW, Shakeshaft A, D’Este C, Green LW (2007). The multiple baseline design for evaluating population-based research. Am J Prev Med.

[31] Ramsay CR, Matowe L, Grilli R, Grimshaw JM, Thomas RE (2003). Interrupted time series designs in health technology assessment: lessons from two systematic reviews of behavior change strategies. Int J Technol Assess Health Care.

[32] Pape UJ, Millett C, Lee JT, Car J, Majeed A (2013). Disentangling secular trends and policy impacts in health studies: use of interrupted time series analysis. J R Soc Med.

[33] Kontopantelis E, Doran T, Springate DA, Buchan I, Reeves D (2015). Regression based quasi-experimental approach when randomisation is not an option: interrupted time series analysis. BMJ.

[34] Taljaard M, McKenzie JE, Ramsay CR, Grimshaw JM (2014). The use of segmented regression in analysing interrupted time series studies: an example in pre-hospital ambulance care. Implement Sci.

[35] Wong RH, Smieliauskas F, Pan I, Lam SK (2015). Interrupted time-series analysis: studying trends in neurosurgery. Neurosurg Focus.

[36] Humphreys DK, Gasparrini A, Wiebe DJ (2017). Evaluating the impact of Florida’s “stand your ground” self-defense law on homicide and suicide by firearm. JAMA. Intern Med.

[37] Bastuji-Garin S, Sbidian E, Gaudy-Marqueste C, Ferrat E, Roujeau JC, Richard MA (2013). Impact of STROBE statement publication on quality of observational study reporting: interrupted time series versus before-after analysis. PLoS One.

[38] Rautiainen RH, Ledolter J, Sprince NL, Donham KJ, Burmeister LF, Ohsfeldt R (2005). Effects of premium discount on workers’ compensation claims in agriculture in Finland. Am J Ind Med.

[39] Fox J. Time-series regression and generalized least squares. An R S-PLUS Companion to Appl. Regres. Thousand Oaks, CA; 2002. p. 1–8.

[40] Jebb AT, Tay L, Wang W, Huang Q (2015). Time series analysis for psychological research: examining and forecasting change. Front Psychol.

[41] Cowpertwait PSP (2009). Metcalfe a V. Introductory time series with R. Gentleman R, Hornik K, Parmigiani G, editors. Media.

[42] Jebb AT, Tay L (2017). Introduction to time series analysis for organizational research. Organ Res Methods.

[43] R Core Team. R: A Language and Environment for Statistical Computing [Internet]. Vienna, Austria: R Foundation for Statistical Computing; 2017. Available from: https://www.r-project.org/.

[44] RStudio Team. RStudio: integrated development for R. Boston, MA: RStudio, Inc.; 2016.

[45] Pinheiro J, Bates D, DebRoy S, Sarkar D, R Core Team. nlme: Linear and Nonlinear Mixed Effects Models [Internet]. 2017. Available from: https://cran.r-project.org/package=nlme

[46] Rimmer SJ, Wilson S (1996). Compliance costs of taxation in Australia.

[47] WorkCoverSA. WorkCoverSA Annual Report 2009–10. Adelaide, AU; 2010.

[48] Bickerdyke I, Lattimore R (1997). Reducing the regulatory burden: does firm size matter?.

[49] McDonald T, Morling S (2011). The Australian economy and the global downturn [internet].

[50] Guthrie R, Aurbach R, Fronsko A (2010). Workers’ compensation and economic downturn: predictions and reflections. Int J Soc Secur Work Compens.

[51] Safe Work Australia. Comparison of Workers’ Compensation Arrangements in Australia and New Zealand 2008. Safe Work Aust. Canberra, AU; 2008.

