# Supplementary material for: Effectiveness of employer financial incentives in reducing time to report worker injury: an interrupted time series study of two Australian workers’ compensation jurisdictions
Source: BMC Public Health. 2018 Jan 5;18:100. doi: 10.1186/s12889-017-4998-9 (PMC5755285; doi:10.1186/s12889-017-4998-9)
Supplement: Supplementary file 1 — Responses to the Quality Criteria for ITS Designs in Ramsay et al. 2003 [31]. Word document with table, citations, and bibliography. (DOCX 24 kb) [file 12889_2017_4998_MOESM1_ESM.docx]

**Supplementary Table 1: Responses to the Quality Criteria for ITS Designs in Ramsay et al. 2003** (1)

| Quality criteria | Response | | | | |
| --- | --- | --- | --- | --- | --- |
| 1. Intervention occurred independently of other changes. | No. Early reporting incentives occurred in the midst of the Global Financial Crisis, which changed claiming behaviour. This was partly controlled for with the use of comparator jurisdictions. However, each jurisdiction introduced its early reporting incentives as part of larger workers’ compensation legislative amendment packages that included many new policies (see Table 1 for a partial list). Findings suggest these may have had an impact on the claims process, including reduction of worker reporting time in SA despite it not being a target (possibly due to provisional liability) and increases in insurer decision time. | | | | |
| 1. Intervention was unlikely to affect data collection. | Yes. Routine WC claims data collection was uninterrupted by early reporting incentives. Coding standards in some WC jurisdictions changed in the study period but not in South Australia, Tasmania, or any jurisdiction in the comparator. However, we cannot rule out the possibility of minor changes to collection practice. | | | | |
| 1. The primary outcome was assessed blindly or was measured objectively. | Yes. The outcomes were objective: median number of days between key events in the claim lodgement process, calculated via date subtraction from administrative WC claims data. | | | | |
| 1. The primary outcome was reliable or was measured objectively. | Yes. The outcomes were objective. See item 3. | | | | |
| 1. The composition of the data set at each time point covered at least 80% of the total number of participants in the study. | Yes. Analyses were limited to jurisdictions with at least 80% usable data (not missing and logical [i.e., not negative]). Tasmania was missing data for 75.6% of its worker report dates and New South Wales for 100%, which resulted in large amounts of missing data for worker and employer reporting time. Thus, they were excluded for analyses on these outcomes, Tasmania as a treatment group and New South Wales as part of the comparator. All other jurisdictions reached the 80% threshold for other dates. The table below reports the number and proportion of incomplete data for each group. | | | | |
|  |  | Claim reporting time | Worker reporting time (excluding New South Wales from comparator and Tasmania and New South Wales from total) | Employer reporting time (excluding New South Wales from comparator and Tasmania and New South Wales from total) | Insurer decision time |
|  |  | Missing (%) | Missing (%) | Missing (%) | Missing (%) |
|  | SA (*n* = 164,656) | 2 (< 0.1%) | 7,591 (4.6%) | 10,530 (6.4%) | 120 (< 0.1%) |
|  | Tasmania (*n* = 54,765) | 1 (< 0.1%) | 41,388 (75.6%) | 41,617 (76.0%) | 1,925 (3.5%) |
|  | Comparator (*n* = 1,250,882;  excluding New South Wales: *n* = 508,945) | 5 (< 0.1%) | 7,807 (1.5%) | 30,175 (5.9%) | 67,253 (5.4%) |
|  | Total (*n* = 1,470,303;  excluding Tasmania and New South Wales: *n* = 508,945) | 8 (< 0.1%) | 15,398 (3.0%) | 40,705 (8.0%) | 69,298 (4.7%) |
| 1. The shape of the intervention effect was pre-specified. | Yes. Early reporting incentives were defined by legislation and designed to reduce total time in the claim lodgement process by reducing employer reporting time. | | | | |
| 1. A rationale for the number and spacing of data points was described. | Yes. We selected a number of data points that would exceed the minimum recommendation, which is usually 16 to 22 (2) or 24 when evaluating seasonal trends in monthly data (3), which would also provide several years of data to better identify and correct for seasonality and autocorrelation. The minimum number of data points pre-ERI was 30 and minimum post-ERI was 24. | | | | |
| 1. The study was analysed appropriately using time series techniques. | Yes. The time series were analysed using a generalised least squares regression (4), accounting for seasonality using harmonic terms (six sine and six cosine, tested and retained only if significant) (5,6), and autoregression (AR) and moving average terms (MA) in ARMA models based on correlated residuals observed in Autocorrelation Function (ACF) and Partial Autocorrelation Function (PACF) plots (3,4,7). | | | | |

# References

1. Ramsay CR, Matowe L, Grilli R, Grimshaw JM, Thomas RE. Interrupted time series designs in health technology assessment: lessons from two systematic reviews of behavior change strategies. Int J Technol Assess Health Care. 2003;19(4):613–23.

2. Penfold RB, Zhang F. Use of interrupted time series analysis in evaluating health care quality improvements. Acad Pediatr. 2013;13(6 SUPPL.):S38–44.

3. Wagner AK, Soumerai SB, Zhang F, Ross-Degnan D. Segmented regression analysis of interrupted time series studies in medication use research. J Clin Pharm Ther. 2002;27(4):299–309.

4. Fox J. Time-series regression and generalized least squares. In: An R and S-PLUS Companion to Applied Regression. Thousand Oaks, CA; 2002. p. 1–8.

5. Jebb AT, Tay L, Wang W, Huang Q. Time series analysis for psychological research: examining and forecasting change. Front Psychol. 2015;6:1–24.

6. Cowpertwait PSP, Metcalfe A V. Introductory Time Series with R. Gentleman R, Hornik K, Parmigiani G, editors. Media. New York, NY: Springer; 2009. 254 p.

7. Jebb AT, Tay L. Introduction to time series analysis for organizational research. Organ Res Methods. 2017;20(1):61–94.
