# Supplementary figures and images for: Effectiveness of employer financial incentives in reducing time to report worker injury: an interrupted time series study of two Australian workers’ compensation jurisdictions
Source: BMC Public Health. 2018 Jan 5;18:100. doi: 10.1186/s12889-017-4998-9 (PMC5755285; doi:10.1186/s12889-017-4998-9)

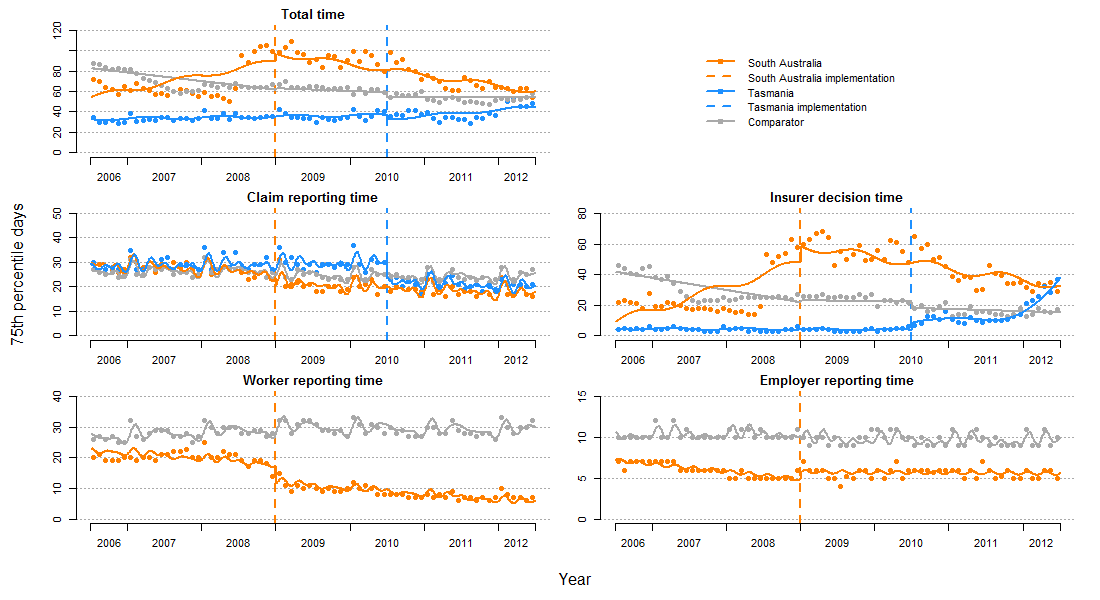

Supplement: Supplementary file 7 — Seasonally-adjusted trends in monthly 75th percentile time lags in the claims process pre- and post-early reporting incentives in South Australia and Tasmania, in reference to a comparator consisting of other Australian workers’ compensation jurisdictions, July 2006 to June 2012. .png image containing plots. (PNG 20 kb) [file 12889_2017_4998_MOESM7_ESM.png]

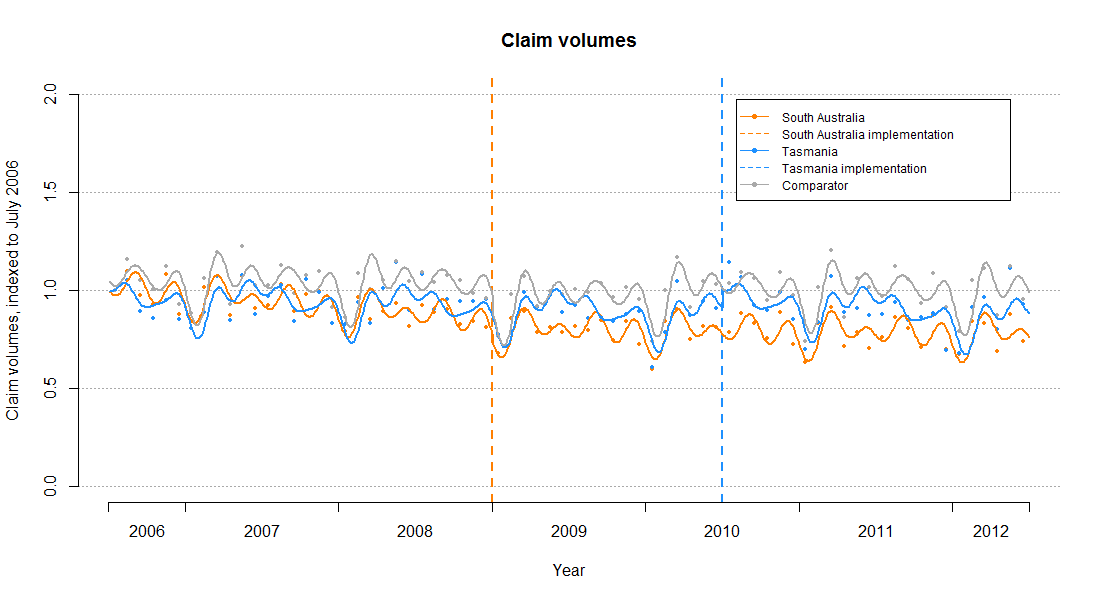

Supplement: Supplementary file 10 — Seasonally-adjusted trends in monthly claim volumes (indexed to July 2006), pre- and post-early reporting incentives in South Australia and Tasmania, in reference to a comparator consisting of other Australian workers’ compensation jurisdictions, July 2006 to June 2012. .png image containing plot. (PNG 14 kb) [file 12889_2017_4998_MOESM10_ESM.png]
