# Supplementary material for: Effectiveness of employer financial incentives in reducing time to report worker injury: an interrupted time series study of two Australian workers’ compensation jurisdictions
Source: BMC Public Health. 2018 Jan 5;18:100. doi: 10.1186/s12889-017-4998-9 (PMC5755285; doi:10.1186/s12889-017-4998-9)
Supplement: Supplementary file 8 — The impact of South Australia and Tasmania’s early reporting incentives on number of days in the claims process, in reference to a comparator consisting of other Australian workers’ compensation jurisdictions (sensitivity analysis with 75th percentile). Word document with table. (DOCX 15 kb) [file 12889_2017_4998_MOESM8_ESM.docx]

**Supplementary Table 2. The impact of South Australia and Tasmania’s early reporting incentives on number of days in the claims process, in reference to a comparator consisting of other Australian workers’ compensation jurisdictions (sensitivity analysis with 75^th^ percentile)**

|  | **ITS analyses** | | | |
| --- | --- | --- | --- | --- |
|  | 75^th^ percentile level change, days (95% CI) | | 75^th^ percentile trend change, days per month (95% CI) | |
| Total time |  |  |  |  |
| South Australia | -0.7 | (-4.7 to 3.3) | -0.36** | (-0.63 to -0.09) |
| Comparator | -0.3 | (-2.5 to 3.2) | 0.11 | (-0.08 to 0.31) |
|  |  |  |  |  |
| Tasmania | -0.7 | (-9.7 to 8.2) | -0.06 | (-0.91 to 0.80) |
| Comparator | -4.1 | (-10.5 to 2.2) | 0.53 | (-0.08 to 1.13) |
|  |  |  |  |  |
| Claim reporting time |  |  |  |  |
| South Australia | -2.0* | (-3.7 to -0.3) | 0.06 | (-0.02 to 0.15) |
| Comparator | -2.0** | (-3.2 to -0.8) | -0.03 | (-0.10 to 0.03) |
|  |  |  |  |  |
| Tasmania | -7.0*** | (-9.1 to -4.9) | -0.20** | (-0.33 to -0.07) |
| Comparator | -0.3 | (-1.9 to 1.2) | 0.06 | (-0.04 to 0.15) |
|  |  |  |  |  |
| Insurer decision time |  |  |  |  |
| South Australia | 5.0 | (-8.3 to 18.3) | -2.34*** | (-3.65 to -1.03) |
| Comparator | 3.1 | (-6.4 to 12.6) | 0.48 | (-0.44 to 1.41) |
|  |  |  |  |  |
| Tasmania^1^ | 3.1 | (-3.6 to 9.9) | -1.73 | (-0.65 to 4.12) |
| Comparator | -3.3 | (-7.5 to 0.9) | 0.44 | (-0.04 to 0.92) |
|  |  |  |  |  |
| Worker reporting time |  |  |  |  |
| South Australia | -8.8*** | (-10.6 to -7.0) | 0.06 | (-0.02 to 0.15) |
| Comparator | 0.2 | (-1.1 to 1.5) | -0.13*** | (-0.19 to -0.07) |
|  |  |  |  |  |
| Employer reporting time |  |  |  |  |
| South Australia | 1.4*** | (0.7 to 2.2) | 0.08*** | (0.04 to 0.12) |
| Comparator | -0.6* | (-1.1 to -0.1) | -0.01 | (-0.04 to 0.02) |

*** p < .001; ** p < .01; p < .05

^1^ Includes squared and cubed time terms to account for non-linearity in post-ERI trend. The model with both squared and cubed trend terms performed significantly better than those without (*p* < .001). Summary statistics: squared trend: -0.24* (-0.46 to -0.03); cubed trend: 0.01** (0.00 to 0.01)
