# Supplementary material for: Effectiveness of employer financial incentives in reducing time to report worker injury: an interrupted time series study of two Australian workers’ compensation jurisdictions
Source: BMC Public Health. 2018 Jan 5;18:100. doi: 10.1186/s12889-017-4998-9 (PMC5755285; doi:10.1186/s12889-017-4998-9)
Supplement: Supplementary file 9 — Monthly counts of workers’ compensation claims pre- and post-early reporting incentives in South Australia and Tasmania, in reference to a comparator consisting of other Australian workers’ compensation jurisdictions, July 2006 to June 2012. Word document with table. (DOCX 15 kb) [file 12889_2017_4998_MOESM9_ESM.docx]

**cSupplementary Table 3: Monthly counts of workers’ compensation claims pre- and post-early reporting incentives in South Australia and Tasmania, in reference to a comparator consisting of other Australian workers’ compensation jurisdictions, July 2006 to June 2012**

|  | **Median monthly claim count (IQR)** | | | | **ITS analyses, excluding comparator** | | | | **ITS analyses, including comparator** | | | |
| --- | --- | --- | --- | --- | --- | --- | --- | --- | --- | --- | --- | --- |
|  | Pre-ERI median (IQR) | | Post-ERI median (IQR) | | Level change, % change from volume indexed at July 2006 (95% CI) | | Trend change, % per month (95% CI) | | Level change, % change from volume indexed at July 2006 (95% CI) | | Trend change, % per month (95% CI) | |
| South Australia | 2,473 | (2,312 to 2,679) | 2,159 | (1,971 to 2,277) | -4.0%** | (-6.7 to -1.2%) | 0.51%*** | (0.37 to 0.65%) | 1.0% | (-2.3 to 4.3%) | 0.30%*** | (0.15 to 0.46%) |
| Comparator | 17,980 | (16,990 to 18,727) | 17,202 | (16,323 to 18,190) | - |  | - |  | -4.8%*** | (-7.1 to -2.4%) | 0.21%*** | (0.10 to 0.32%) |
|  |  |  |  |  |  |  |  |  |  |  |  |  |
| Tasmania | 754 | (714 to 814) | 743 | (710 to 789) | 9.2%* | (1.6 to 16.8%) | -0.32% | (-0.78 to 0.15%) | -0.3% | (-7.8 to 7.3%) | -0.17% | (-0.63 to 0.28%) |
| Comparator | 17,530 | (16,727 to 18,423) | 17,629 | (16,250 to 18,654) | - |  | - |  | 8.3%** | (2.9 to 13.6%) | -0.09% | (-0.42 to 0.23%) |

*** p < .001; ** p < .01; p < .05
